# Retraction: Nogo-C inhibits peripheral nerve regeneration by regulating Schwann cell apoptosis and dedifferentiation

**DOI:** 10.3389/fnins.2025.1672559

**Published:** 2025-07-30

**Authors:** Frontiers Editorial Office

The publisher retracts the article cited above.

Following publication, the authors requested the retraction of the paper. Further concerns were then raised regarding the integrity of the images in the published figures. The authors failed to provide a satisfactory explanation during the investigation, which was conducted in accordance with Frontiers' policies.

This retraction was approved by the Chief Executive Editor of Frontiers. The authors agree to this retraction.

Frontiers would also like to thank Sholto David for bringing the published article to our attention.

